# Anti-C5a antibody vilobelimab treatment and the effect on biomarkers of inflammation and coagulation in patients with severe COVID-19: a substudy of the  phase 2 PANAMO trial

**DOI:** 10.1186/s12931-022-02278-1

**Published:** 2022-12-24

**Authors:** Endry H. T. Lim, Alexander P. J. Vlaar, Lieuwe D. J. Bos, Lonneke A. van Vught, Anita M. Tuip-de Boer, Romein W. G. Dujardin, Maria Habel, Zhongli Xu, Matthijs C. Brouwer, Diederik van de Beek, Sanne de Bruin, the Amsterdam UMC COVID-19 Biobank Investigators, Michiel van Agtmael, Anne Geke Algera, Brent Appelman, Floor van Baarle, Martijn Beudel, Harm Jan Bogaard, Marije Bomers, Peter Bonta, Lieuwe D. J. Bos, Michela Botta, Justin de Brabander, Godelieve Bree, Marianna Bugiani, Esther Bulle, Osoul Chouchane, Alex Cloherty, David T. P. Buis, Maurits C. F. J. de Rotte, Mirjam Dijkstra, Dave A. Dongelmans, Paul Elbers, Lucas Fleuren, Suzanne Geerlings, Theo Geijtenbeek, Armand Girbes, Bram Goorhuis, Martin P. Grobusch, Laura Hagens, Jorg Hamann, Vanessa Harris, Robert Hemke, Sabine M. Hermans, Leo Heunks, Markus Hollmann, Janneke Horn, Joppe W. Hovius, Menno D. de Jong, Rutger Koning, Niels van Mourik, Jeannine Nellen, Esther J. Nossent, Frederique Paulus, Edgar Peters, Dan A. I. Piña-Fuentes, Tom van der Poll, Bennedikt Preckel, Jan M. Prins, Jorinde Raasveld, Tom Reijnders, Michiel Schinkel, Femke A. P. Schrauwen, Marcus J. Schultz, Alex Schuurman, Jaap Schuurmans, Kim Sigaloff, Marleen A. Slim, Patrick Smeele, Marry Smit, Cornelis S. Stijnis, Willemke Stilma, Charlotte Teunissen, Patrick Thoral, Anissa M. Tsonas, Pieter R. Tuinman, Marc van der Valk, Denise Veelo, Carolien Volleman, Heder de Vries, Michèle van Vugt, Dorien Wouters, Aeilko H. Zwinderman, W. Joost Wiersinga

**Affiliations:** 1 Department of Intensive Care Medicine, Amsterdam UMC Location University of Amsterdam, Meibergdreef 9, Amsterdam, The Netherlands; 2 Laboratory of Experimental Intensive Care and Anaesthesiology (L.E.I.C.A.), Amsterdam, The Netherlands; 3 Department of Neurology, Amsterdam UMC Location University of Amsterdam, Meibergdreef 9, Amsterdam, The Netherlands; 4 Amsterdam Neuroscience, Amsterdam, The Netherlands; 5 Center for Experimental and Molecular Medicine, Amsterdam UMC Location University of Amsterdam, Amsterdam, The Netherlands; 6 InflaRx GmbH, Jena, Germany; 7 Department of Intensive Care Medicine, Amsterdam UMC, Location AMC, Room C3-421, Meibergdreef 9, 1105 AZ Amsterdam, The Netherlands

**Keywords:** SARS-CoV-2, COVID-19, Complement, Complement inhibition, Vilobelimab, C5a

## Abstract

We recently reported in the phase 3 PANAMO trial that selectively blocking complement 5a (C5a) with vilobelimab led to improved survival in critically ill COVID-19 patients. C5a is an important contributor to the innate immune system and can also activate the coagulation system. High C5a levels have been reported in severely ill COVID-19 patients and correlate with disease severity and mortality. Previously, we assessed the potential benefit and safety of vilobelimab in severe COVID-19 patients. In the current substudy of the phase 2 PANAMO trial, we aim to explore the effects of vilobelimab on various biomarkers of inflammation and coagulation. Between March 31 and April 24, 2020, 17 patients with severe COVID-19 pneumonia were enrolled in an exploratory, open-label, randomised phase 2 trial. Blood markers of complement, endothelial activation, epithelial barrier disruption, inflammation, neutrophil activation, neutrophil extracellular trap (NET) formation and coagulopathy were measured using enzyme-linked immunosorbent assay (ELISA) or utilizing the Luminex platform. During the first 15 days after inclusion, change in biomarker concentrations between the two groups were modelled with linear mixed-effects models with spatial splines and compared. Eight patients were randomized to vilobelimab treatment plus best supportive care (BSC) and nine patients were randomized to BSC only. A significant decrease over time was seen in the vilobelimab plus BSC group for C5a compared to the BSC only group (p < 0.001). ADAMTS13 levels decreased over time in the BSC only group compared to the vilobelimab plus BSC group (p < 0.01) and interleukin-8 (IL-8) levels were statistically more suppressed in the vilobelimab plus BSC group compared to the BSC group (p = 0.03). Our preliminary results show that C5a inhibition decreases the inflammatory response and hypercoagulability, which likely explains the beneficial effect of vilobelimab in severe COVID-19 patients. Validation of these results in a larger sample size is warranted.

**Supplementary Information:**

The online version contains supplementary material available at 10.1186/s12931-022-02278-1.

## Introduction

We recently reported positive results from the multicentre, double-blind, randomised, placebo-controlled, phase 3 PANAMO trial in which we targeted complement 5a (C5a) with vilobelimab, an anti-C5a monoclonal antibody, in critically ill, invasively mechanically ventilated patients with COVID-19 [1]. The addition of vilobelimab to best supportive care (BSC) improved survival and led to a significant decrease in mortality [1]. C5a is an important contributor to the innate immune system and has the potency to activate the coagulation system [2, 3]. It is a strong chemoattractant for neutrophils and it plays a role in the recruitment of inflammatory cells such as monocytes and macrophages. This may not only contribute to innate immune functions, but also causes tissue damage [2]. Additionally, C5a has been shown to activate the coagulation pathways, either indirectly via C5a-elicited inflammation or injury of endothelial cells, or directly by the induction of tissue factor expression on endothelial cells [2, 4–6].

High C5a levels are a hallmark of severe COVID-19, due to a direct increased activation of the complement system by SARS-CoV-2 [7–10]. Increased C5a levels are associated with endothelial injury, hypercoagulation and increased inflammation, ultimately contributing to poor outcomes [7, 10, 11]. This could be the result of abundant recruitment and activation of neutrophils, monocytes and macrophages, which have been suggested to play a central role in the pathogenesis of severe COVID-19 [10, 12]. Subsequently, complement activation and neutrophil extracellular traps (NETs) have been identified as key drivers in COVID-19 immunothrombosis, a characteristic feature in severe COVID-19 patients [13, 14]. Accordingly, C5a levels have been demonstrated to correlate with disease severity and mortality [7].

Therefore, inhibition of C5a may attenuate disease severity in severely ill COVID-19 patients and improve outcomes. We previously assessed the potential benefit and safety of selectively blocking C5a in severe COVID-19 patients with vilobelimab in the phase 2 PANAMO trial [15]. Secondary outcomes such as mortality at day 28, kidney function and proportion of pulmonary embolisms classified as serious appeared to be in favour of vilobelimab treatment. Here, we report a substudy of the phase 2 PANAMO trial in which we aim to explore the effect of vilobelimab on a various biomarkers of inflammation and coagulation.

## Methods

### Study design

For the current analysis, results from two individual studies were combined that included the same set of patients. Clinical data and biomarker measurements were used from patients in the phase 2 PANAMO trial, who were included in the academic hospital Amsterdam UMC, location AMC. The phase 2 PANAMO trial is an exploratory, open-label, multicentre, randomised phase 2 trial in patients with severe COVID-19 [15]. Patients were randomised 1:1 between vilobelimab plus BSC or BSC only. Vilobelimab was administered on days 1, 2, 4, 8, 15 and optionally on days 11–13. An additional dose was administered to patients who were still intubated on day 22. Randomisation was stratified by study site. Patients with an age of ≥ 18 years and severe COVID-19 pneumonia were included. Severe COVID‐19 was defined as severe pneumonia with pulmonary infiltrates consistent with pneumonia, a clinical history of severe shortness of breath within the past 14 days, or a need for non-invasive or invasive ventilation; severe disease was defined as a ratio of partial pressure of arterial oxygen to fractional concentration of oxygen in inspired air (PaO_2_/FiO_2_) between 100 and 250 mmHg in the supine position [15]. Patients were included between March 31 until April 24, 2020, and follow-up time was until 28 days after inclusion. The protocol was approved by the medical research ethics committee of the Amsterdam UMC, location AMC (IRB 2020_067#B2020179).

Additional biomarker data and clinical characteristics of all the patients included in the phase 2 PANAMO trial at Amsterdam UMC, location AMC, were extracted from the Amsterdam UMC COVID-19 Biobank study. In this study, all consecutive COVID-19 patients admitted to the ward or intensive care unit in the Amsterdam UMC from March 23 to May 26, 2020, were included [9]. Clinical data and daily left over plasma were collected and stored in the Amsterdam UMC COVID-19 biobank for future research questions. This study was approved by the biobank ethics committees of Amsterdam UMC (2020_065).

### Plasma protein biomarker measurement

For all patients included in the phase 2 PANAMO trial, EDTA plasma was collected up to seven times during treatment, one hour prior to vilobelimab administration or on the corresponding study day for the control group. Samples were centrifuged at 2000×*g* and stored at − 80 °C. Enzyme-linked immunosorbent assays (ELISA) were used to measure thromboxane A2 (R&D Systems), and complement markers factor Bb (Quidel), MASP-2 (MyBiosource), C3a (Quidel) and C5a (InflaRx’ in-house developed and validated ELISA). C3a and C5a were also measured earlier, as part of the phase 2 PANAMO trial, in another laboratory using the same samples [16].

Additional samples were selected from the Amsterdam UMC COVID-19 Biobank study for plasma protein biomarkers of endothelial activation, epithelial barrier disruption, inflammation, neutrophil activation, NET formation and coagulopathy [9]. These biomarkers were measured in heparin plasma longitudinally, at several time points after admission to the hospital, using an ELISA or the Luminex platform, as previously described [9]. Only data obtained during the inclusion period of the phase 2 PANAMO trial was used for this substudy.

### Endpoints

The primary aim was to explore the difference in biomarker concentrations over time between patients treated with vilobelimab plus BSC, and patients treated with BSC only.

### Statistics

Normally distributed data are expressed as mean (SD) and non-normally distributed data as median (IQR). Normality of data was assessed visually and by using the Shapiro–Wilk test. Differences between the two groups were assessed with a t-test or Mann–Whitney U test as appropriate. Biomarker concentrations were log-transformed in order to normalize the distribution. In both groups, changes in biomarker concentration during the first 15 days after inclusion were modelled with linear mixed-effects models (LMMs). LMMs with spatial splines were used due to non-linearity of the data. A fixed effect interaction term was used between study day and randomization group, with patient identification number as random intercept. The model without the randomization factor and the model with the randomization factor as interaction terms were compared using the Likelihood Ratio Test. Sampling beyond day 15 was considered too sparse to be representative. Correction for multiple testing was not performed due to the exploratory character of the study and a relatively low sample size [17]. All statistical analyses were performed in R-Studio (version 4.0.3; Boston, MA). P-value for statistical significance was set at 0.05 for all analyses.

## Results

### Patient characteristics

From March 31, 2020, through April 24, 2020, eight patients were randomized to vilobelimab treatment plus BSC and nine patients to BSC only. Baseline characteristics were comparable between the two groups (Table 1). All patients in the vilobelimab plus BSC group received a minimum of four vilobelimab infusions. Two patients (25%) received four infusions, two patients (25%) received five infusions, two patients (25%) received six infusions and two patients (25%) received seven infusions. Patients who did not receive all planned infusions either died or were discharged from the hospital before all planned infusions were administered.

**Table 1:** Baseline characteristics at randomization

	Vilobelimab + BSC	BSC
Characteristics		
n	8	9
Sex = male (%)	5 (62.5)	6 (66.7)
Age	61.5 [51.0, 61.5]	61.0 [55.0, 68.0]
Body-mass index	27.3 [24.4, 31.1]	29.0 [25.8, 30.6]
Race (%)		
Asian	4 (50.0)	2 (22.2)
Black or African American	2 (25.0)	2 (22.2)
White	2 (25.0)	5 (55.6)
Days since onset of COVID-19 symptoms	11.00 [7.5, 11.5]	13.0 [12.0, 14.0]
Medical history		
Asthma or other chronic pulmonary disease	2 (25.0)	0 (0.0)
Cardiac disorders	3 (37.5)	1 (11.1)
Diabetes mellitus type 2	3 (37.5)	2 (22.2)
Hypertension	4 (50.0)	2 (22.2)
Obesity	2 (25.0)	4 (44.4)
Number of comorbidities	2.5 [1.0, 3.3]	2.0 [1.0, 2.0]
Patients with relevant comorbidities	7 (87.5)	5 (55.6)
Disease severity		
Intubated at randomization	6 (75.0)	8 (88.9)
On ICU at randomization	6 (75.0)	8 (88.9)
qSOFA	1.0 [0.8, 1.3]	1.0 [1.0, 2.0]
MEWS	3.5 [2.0, 5.3]	4.0 [4.0, 6.0]
Previous medication		
Antibiotics	8 (100.0)	7 (77.8)
Chloroquine	1 (12.5)	2 (22.2)
Remdesivir	0 (0.0)	0 (0.0)
Steroids	2 (25.0)	1 (11.1)

*BSC* best supportive care, *MEWS* Modified Early Warning Score, *qSOFA* quick Sequential Organ Failure Assessment

### Effect of vilobelimab on biomarker levels

**Fig. 1:**
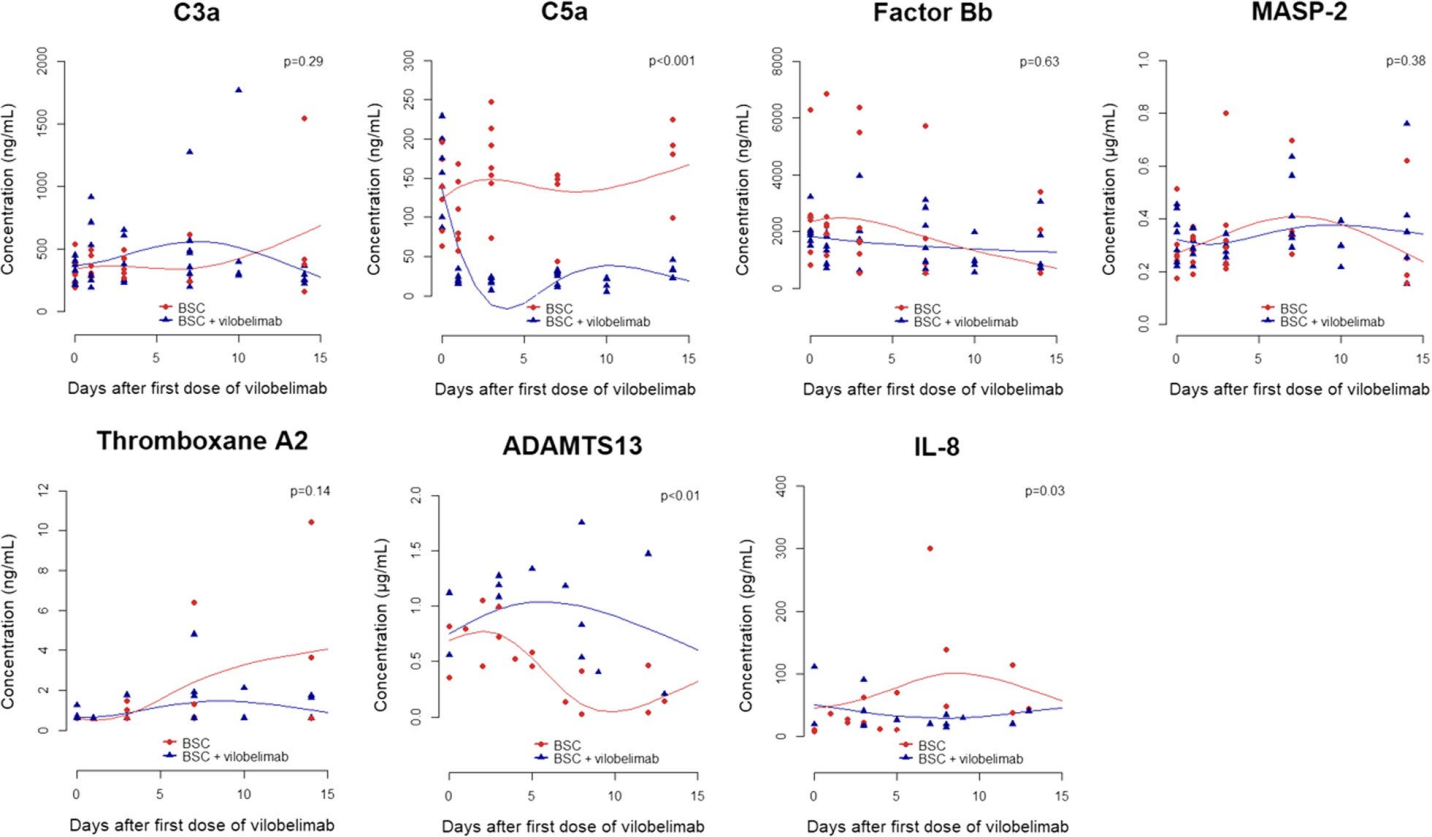
C3a, C5a, factor Bb, MASP-2, thromboxane A2, ADAMTS13 and IL-8. Mean predictions of the LMMs per group are plotted in the graphs. *ADAMTS13* a disintegrin and metalloproteinase with a thrombospondin type 1 motif, member 13, *BSC* best supportive care, *IL-8* interleukin-8, *MASP-2* Mannan-binding lectin serine protease 2

Baseline C5a concentrations were elevated compared to healthy subjects and comparable between the two groups, 156.4 ng/ml [119.7, 187.3] in the vilobelimab plus BSC group and 139.1 ng/ml [103.0, 185.2] in the BSC only group [16]. After one infusion, median C5a levels were 19.3 ng/ml [16.3, 23.5] in the vilobelimab plus BSC group compared to 95.5 ng/ml [74.8, 136.8] in the BSC only group (p = 0.002). A significant decrease over time was seen in the vilobelimab plus BSC group for C5a compared to the BSC only group (p < 0.001) (Fig. 1). The mean predictions of the LMMs per group are plotted for each biomarker to demonstrate the fit of the model. Biomarker concentrations did not differ significantly for complement markers C3a, MASP-2, factor Bb and thromboxane A2 (Fig. 1). Although sampling was scarce, ADAMTS13 levels decreased over time in the BSC only group compared to the vilobelimab plus BSC group (p < 0.01) and interleukin-8 (IL-8) levels appeared to be more suppressed in the vilobelimab plus BSC group (p = 0.03) (Fig. 1). C5a, ADAMTS13 and IL-8 remained significantly different between the two groups when tested over 28 days. Other blood protein plasma markers of endothelial activation, epithelial barrier disruption, inflammation, neutrophil activation and NET formation, complement markers and coagulopathy were comparable between the two groups.

## Discussion

In this exploratory substudy of the phase 2 PANAMO study, we evaluated the effect of vilobelimab on various biomarkers. Our results confirm that C5a levels are elevated in severe COVID-19 patients and that vilobelimab significantly decreased C5a levels over 15 days compared to the BSC only group. As expected, biomarker concentrations did not differ significantly for complement markers C3a, MASP-2 and factor Bb as these complement factors are upstream of C5a, which is directly inhibited by vilobelimab. Thromboxane A2 concentrations did not differ significantly between the two groups either. Although sampling was scarce, ADAMTS13 levels decreased over time in the BSC only group compared to the vilobelimab plus BSC group and IL-8 levels appeared to be more suppressed in the vilobelimab plus BSC group. These results can give a first suggestion as to why inhibition of C5a is beneficial in severely ill COVID-19 patients.

In addition to proinflammatory properties of C5a, the ability to activate the coagulation system [7] was further demonstrated by the stable ADAMTS13 levels in the intervention group. ADAMTS13 is an enzyme which primarily functions to cleave von Willebrand factor (VWF) multimers on endothelial surfaces, in the circulation and at the sites of vascular injury [18]. VWF is an adhesive and multimeric glycoprotein which mediates platelet adhesion to injured vascular subendothelium and platelet aggregation [19]. Large VWF multimers are prothrombotic, thus tight regulation by ADAMTS13 is essential [19]. Compared to the vilobelimab plus BSC group, ADAMTS13 levels decreased significantly in the BSC only group, most likely as a result of consumption. This suggests that C5a inhibition may mitigate COVID-19 associated endotheliopathy, thereby possibly protecting against thrombotic complications in COVID-19 [20]. In line with this, an elevated von Willebrand factor antigen (VWF:Ag) to ADAMTS13 activity ratio was strongly associated with disease severity in a cross-sectional study, which increases the hypercoagulable state of COVID-19 patients and the risk of microthrombosis [20]. Unfortunately, in our study VWF samples were too sparse to be representative.

Levels of IL-8, which can be induced by C5a [21], appeared to be decreased in the vilobelimab plus BSC treated group compared to the BSC only group. IL-8 is a proinflammatory chemokine and has an important role in the activation of neutrophils. It plays a significant role in the pathogenesis of ARDS, and increasing evidence points towards a role of IL-8 in COVID-19 as well [22]. Elevated levels of IL-8 were significantly associated with duration of illness in severe COVID-19 patients [23].

Other biomarkers of inflammation, neutrophil activation, NET formation and coagulopathy did not differ significantly over time between the two groups. A major limitation of this study is the low number of patients and scarce sampling as only patients admitted to the Amsterdam UMC, location AMC hospital were included in this substudy. The low number of patients and scarce sampling could have led to type 2 errors due to insufficient power. In addition, sampling of biomarkers of the COVID-19 Biobank study was dependent on clinical blood drawings, since only left-over plasma was collected. Therefore, these results should be considered preliminary and validation of these results in a larger sample size is warranted. An advantage of LMMs is that all measurements are taken into account in the analysis. Furthermore, it remains hypothetical whether C5a inhibition directly leads to the results found, or via attenuation of disease severity in COVID-19 patients.

## Conclusion

In this exploratory sub-study, severely ill COVID-19 patients treated with vilobelimab plus BSC showed a significant decrease of C5a during the first 15 days, compared to the BSC only group. ADAMTS13 levels decreased over time in the BSC only group compared to the vilobelimab plus BSC group, potentially indicating a protective effect of vilobelimab on thrombotic complications. IL-8 levels, which can be induced by C5a, appeared to be more suppressed in patients treated with vilobelimab plus BSC.

Our results provide preliminary data showing C5a inhibition decreases the inflammatory response and hypercoagulability, which is likely to explain the beneficial effect of vilobelimab in severe COVID-19 patients. Validation of these results in a larger sample size is warranted.

## Supplementary Information

**Additional file 1.** Amsterdam UMC COVID-19 Biobank Investigators.

## Data Availability

Data will be shared according to applicable regulatory requirements.
